# Effects of Repetitive Hyperbaric Oxygen Treatment in Patients with Acute Cerebral Infarction: A Pilot Study

**DOI:** 10.1100/2012/694703

**Published:** 2012-08-01

**Authors:** Cheng-Hsin Chen, Shao-Yuan Chen, Vinchi Wang, Chao-Ching Chen, Kaw-Chen Wang, Chih-Hao Chen, Yi-Chien Liu, Kuo-Cheng Lu, Ping-Keung Yip, Wen-Ya Ma, Chuan-Chieh Liu

**Affiliations:** ^1^Department of Internal Medicine, Cardinal Tien Hospital, New Taipei City, Taiwan; ^2^Department of Neurology, Cardinal Tein Hospital, New Taipei City, Taiwan; ^3^Department of Hyperbaric Medicine, Cardinal Tein Hospital, New Taipei City, Taiwan; ^4^Department of Medical Research, Cardinal Tien Hospital, New Taipei City, Taiwan; ^5^School of Medicine, Fu Jen Catholic University, New Taipei City, Taiwan

## Abstract

The role of hyperbaric oxygen therapy (HBOT) in the treatment of acute ischemic stroke is controversial. This prospective study assessed the efficacy and safety of HBOT as adjuvant treatment on 46 acute ischemic stroke in patients who did not receive thrombolytic therapy. The HBOT group (*n* = 16) received conventional medical treatment with 10 sessions of adjunctive HBOT within 3–5 days after stroke onset, while the control group (*n* = 30) received the same treatment but without HBOT. Early (around two weeks after onset) and late (one month after onset) outcomes (National Institutes of Health Stroke Scale, NIHSS scores) and efficacy (changes of NIHSS scores) of HBOT were evaluated. The baseline clinical characteristics were similar in both groups. Both early and late outcomes of the HBOT group showed significant difference (*P* ≤ 0.001). In the control group, there was only significant difference in early outcome (*P* = 0.004). For early efficacy, there was no difference when comparing changes of NIHSS scores between the two groups (*P* = 0.140) but there was statistically significant difference when comparing changes of NIHSS scores at one month (*P* ≤ 0.001). The HBOT used in this study may be effective for patients with acute ischemic stroke and is a safe and harmless adjunctive treatment.

## 1. Introduction

In most developed countries, cerebrovascular disease is always ranked in the top ten causes of death. In Taiwan, about 70% of hospitalized acute stroke patients have ischemic stroke. Although the mortality rate of acute ischemic stroke is less than that of hemorrhagic stroke, it still results in patient disabilities and complications that often lead to significant costs to individuals, families, and society.

Traditional treatment for acute ischemic stroke includes thrombolytic therapy by injecting t-PA within three hours after onset of symptoms, and antiplatelet and/or anticoagulant agents administered within the first 48 hours. Clinically, the narrow time window of thrombolytic therapy and coexisting contraindications limit the use of t-PA [1, 2]. Thus, searching for an effective supplemental treatment for acute ischemic stroke is imperative. 

Hyperbaric oxygen therapy (HBOT) is valuable in treating acute CO poisoning, air or gas embolism [3, 4] and in facilitating wound healing [5]. Known mechanisms of HBOT-induced neuroprotection include enhancing neuronal viability via increased tissue oxygen delivery to the area of diminished blood flow, reducing brain edema, and improving metabolism after ischemia [6, 7]. Furthermore, a recent study performed on a rat suggested that upregulation of the expression of glial derived neurotrophic factor (GDNF) and nerve growth factor (NGF) might underlie the effect of HBOT [8]. Despite beneficial results in several animal models [9, 10], the effectiveness in human ischemic stroke is still controversial. In a randomized, prospective, double-blind, and sham-controlled pilot study published in 2003, Rusyniak et al. delivered HBOT to 33 patients with acute ischemic stroke who did not receive thrombolytic therapy, and assessed the therapeutic effectiveness after 24 hours and 90 days. They concluded that HBOT did not appear to be beneficial and might even be harmful for patients with acute ischemic stroke [11]. 

However, the viewpoint and shortcomings of this trial were challenged by others who suggested that further studies were needed to determine the feasibility of lower pressures, greater numbers of dives with better clinical outcomes, and suitable stroke subtype. In 2006, Bennett et al. included three randomized controlled trials (106 participants) to assess the benefits and safety of adjunctive HBOT in the treatment of acute ischemic stroke. Their systematic review did not demonstrate that HBOT could improve clinical outcomes. However, due to the lack of guidelines for study design, the use of HBOT in stroke patients could not be justified [12]. 

Moreover, a previous report showed a 56-year-old Chinese patient suffering from acute ischemic stroke on the left corona radiata leading to right hemiparesis and dysarthria, had great improvement after repetitive (10 dives) HBOT with lower pressure (2.0 ATA) and short duration (60 min) [13].

This study prospectively assessed the efficacy and feasibility of applying 10 repetitions of HBOT at 2.0 ATA for one hour in patients with mild acute ischemic stroke within 3–5 days after stroke onset to reinvestigate the role of HBOT in treating acute ischemic stroke.

## 2. Methods

This prospective, open-label study was conducted in a local teaching hospital and compared the efficiency of receiving HBOT with traditional therapy for treatment of acute ischemic stroke. This hospital's Institutional Review Board approved the study and all participants provided informed consent.

### 2.1. Patient Selection

Adult (aged >18 years) patients were included if they had a diagnosis of acute ischemic stroke within 48 hours after onset, according to imaging findings by brain computed tomography (CT) without evidence of hemorrhage, upon admission to the hospital. No patient received thrombolytic therapy. Upon admission, the vital signs, personal history (e.g., smoking and alcohol consumption), laboratory data including CBC/DC, electrolytes, and lipid profiles, and past medical history, including type 2 diabetes mellitus, hypertension, or cardiovascular disease were reviewed. 

The severity of disease upon admission was scored using the National Institutes of Health Stroke Scale (NIHSS). “Mild” disease was defined as score of 0–14, “moderate” as 15–28, and “severe” as 29–42. Patients with moderate or severe disease were not eligible, as well as those with contraindications or risk factors for HBOT (i.e., uncontrolled high fever, emphysema with CO_2_ retention, pneumothorax, or seizure disorder). 

### 2.2. Interventions

Subjects were divided into the HBOT group, who received HBO therapy, and the control group, who received similar treatment except HBOT. The HBOT group underwent 10 repetitive treatments for 60 minutes in a hyperbaric chamber pressured with compressed air, whereas patients breathed 100% oxygen to 2 ATA. 

### 2.3. Outcome Measures

As pretreatment evaluation, all patients were evaluated by NIHSS within 48 hours after admission. As posttreatment evaluation, patients in the HBOT group were evaluated by NIHSS after 10 sessions of HBOT. The control group was evaluated with NIHSS 10 days after stroke onset. One month after treatment, all patients were evaluated again using the NIHSS.

### 2.4. Efficacy Evaluations

The clinical response was presented by comparing the patient's pre-treatment NIHSS scores with those evaluated after 10 sessions of HBOT in the HBOT group. Similarly, the NIHSS scores within 48 hours of stroke onset were compared to those 14 days after stroke in control group. Long-term efficacy was determined by comparing the pretreatment NIHSS score with those taken after one month in both groups. 

To determine the HBOT efficacy, changes of NIHSS scores between the HBOT and control groups were demonstrated by (1) early efficacy, or changes in NIHSS scores at the time before HBOT and after 10 sessions of HBOT in HBOT group were compared to changes of scores between the early days and 14 days after stroke onset in the control group, and (2) late efficacy, where changes of NIHSS scores between pretreatment and one month posttreatment in the HBOT group were compared with changes of scores between the early days and one month after stroke onset in the control group.

### 2.5. Statistical Analysis

Data analysis was performed using the SPSS Statistics version 19 software and the figures were constructed by Sigmaplot version 12 software. Baseline categorical variables were analyzed by Fisher's exact test, whereas numerical variables were analyzed by Student's *t*-test (equal variance test). To compare paired data, the Shapiro-Wilk test was used for the HBOT group and the Shapiro-Wilk test and Wilcoxon Signed Rank test were used in the control group to test the data of early and long-term clinical responses. Variables in both groups (e.g., changes of NIHSS scores) were compared by Equal Variance Test for early efficacy and by the Mann-Whitney Rank Sum Test for late efficacy. For all analysis, *P* < 0.05 was considered statistically significant.

## 3. Results

**Figure 1:**
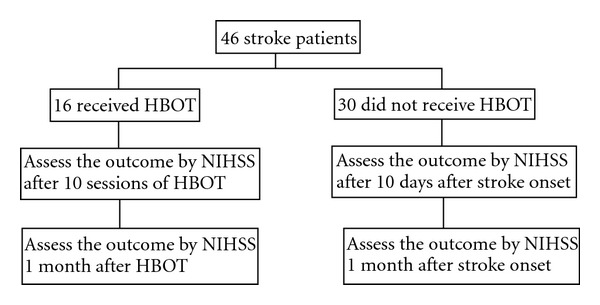
Hyperbaric oxygen therapy in acute ischemic stroke trial.

From February 2007 to April 2010, 46 patients were recruited, including 16 in the HBOT group and 30 in the control group (Figure 1). Their baseline properties and clinical characteristics were compared. Categorical variables analyzed by Fishers exact test and numerical variables analyzed by Student's *t*-test showed no statistically significant difference between the two groups in each item (*P* value ranged from 0.063 to 0.930; Table 1).

All the patients completed the treatment schedule. The HBOT was given with 2 absolute atmospheres (ATA) to all the patients in the HBOT group. Treatments lasted 60 minutes for 10 sessions, once a day, for two weeks. Treatment outcome was evaluated by the NIHSS score immediately after HBOT and at one month after onset of stroke attack. The schedule of testing by the NIHSS score in the control group was the same. 

**Figure 2:**
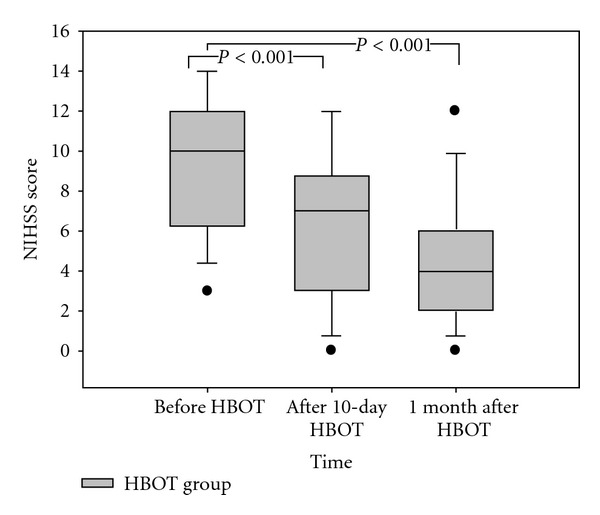
The clinical outcome (NIHSS score) in the HBOT group. The NIHSS scores were significantly decreased immediately after HBOT and after one month compared to the scores before HBOT (*P* < 0.001).

In the HBOT group, the paired *t*-test was used to compare the NIHSS scores before HBOT (mean ± SE 9.313 ± 3.459) with either the 2 weeks post-HBOT scores (6.188 ± 3.920) or the scores at one month after the stroke (4.438 ± 3.245). The NIHSS scores were significantly decreased immediately after HBOT and at one month later compared to the pre-HBOT data (*P* < 0.001) (Figure 2).

**Figure 3:**
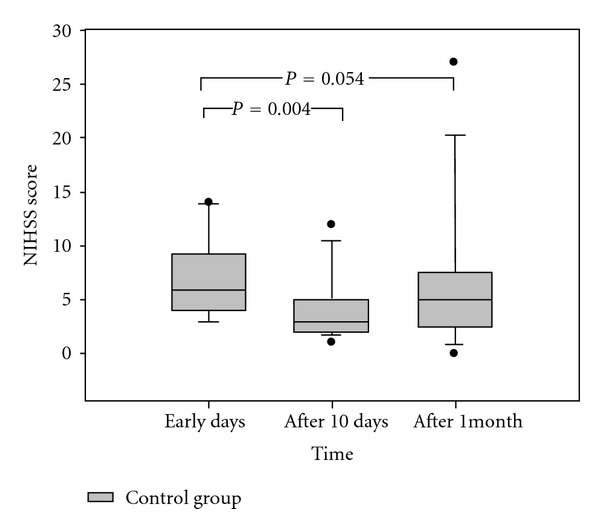
The clinical outcome (NIHSS score) in the control group. The NIHSS scores were significantly decreased on the 10th day after stroke (*P* < 0.05) but without any difference after one month (*P* = 0.054).

In the control group, the scores were evaluated in the same time schedule as the HBOT group. The early outcomes were demonstrated by comparing the scores within early 3–5 days to the scores on the 14th day after stroke onset with paired *t*-test (*P* = 0.004). However, the scores between early and late outcomes were not significantly different (*P* = 0.054) (Figure 3).

**Figure 4:**
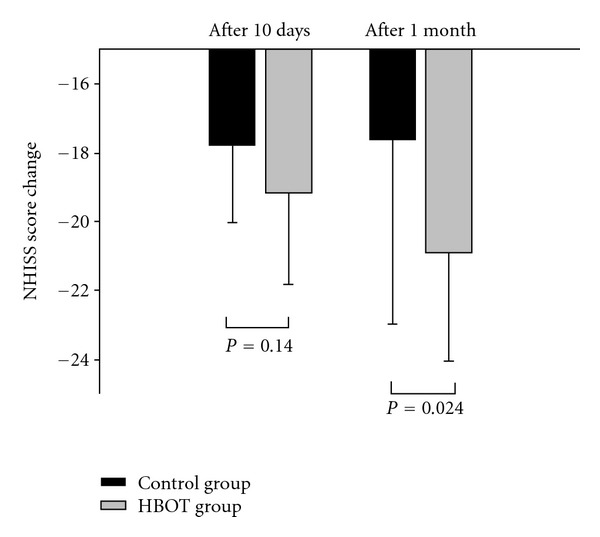
The change of NIHSS scores in both groups. There was no significant NIHSS change on the 10th day after stroke (*P* = 0.140) but there was significant difference after one month (*P* = 0.024).

The difference of basal NIHSS scores between the HBOT group and control group was not statistically significant (*P* = 0.647) and there was no statistically significant difference on early efficacy between the groups (*P* = 0.140). However, comparison of late efficacy showed a statistically significant difference between the groups (*P* = 0.024) (Figure 4). 

## 4. Discussion

The present study demonstrates better clinical outcome evaluated by NIHSS in acute stroke patients with adjunctive HBOT therapy than those without, which is different from results of previous clinical studies. Compared to several successful animal studies in acute ischemic stroke, human studies contain many factors that may influence outcomes, such as age, severity of stroke, social circumstances, prior disability, and comorbidities [14]. Moreover, the time and protocol of HBOT play a role in the prognosis of acute ischemic stroke [15–17]. Compared to other trials wherein HBOT is not beneficial and may instead be harmful for acute ischemic stroke [11], the present study highlights several main points. First, the acute ischemic stroke patients recruited here have initially mild disease severity (NIHSS score 1–14). The confounding effect of disease severity was avoided. Second, HBOT was applied to the stroke patients within 3–5 days after stroke onset because HBOT did not have a significant thrombolytic effect as t-PA. The stroke condition during this period was more stable than it was within 3 hours or 24 hours. 

Third, the protocol of HBOT was using lower pressure (2.0 ATA) and shorter duration (60 mins) to avoid possible oxidative stress caused by high pressure [10, 12, 18]. Lastly, the patients were treated with repetitive HBOT for a total of 10 sessions (once per day for 2 weeks). According to previous studies, HBOT may be associated with several rare adverse effects, including damage to the ears, sinuses and lungs from the effects of pressure, pneumothorax, air embolism, seizure, and oxygen poisoning [12, 19, 20]. In this study, all patients completed the treatment protocol and very few received myringotomy before HBOT for possible middle ear barotraumas. It is reasonable to consider that HBOT is a safe and feasible intervention. 

The present study suggests that HBOT may be effective for acute ischemic stroke patients, and the efficacy of therapy is more obvious later in the disease course. A comparison of the two groups at one month revealed that the HBOT group had better outcome than the control group (*P* = 0.024), although outcome in the early days did not show significant difference (*P* = 0.140). All patients in the HBOT group showed improvement except for one patient who presented with worse outcome after 10 repetitions of HBOT (NIHSS score from 7 to 9), but no more disease progression after one month (NIHSS score from 9 to 9). Although the early efficacy of HBOT did not reach the statistic significance, it did reveal the tendency of better outcome compared to the control group. There are possible explanations. One, the mechanisms of HBOT for ischemic stroke (i.e., protection of the blood-brain barrier) repair and generate new blood vessels to the parts of the brain that have been injured, inhabited by apoptosis, and improve metabolism after ischemia [6]. All of these effects may take time to occur. Two, cerebral edema always develop soon after stroke onset and peak at about 24–96 hours. It decreases blood flow and influence oxygen delivery [21]. Three, since HBOT is an adjunctive treatment for ischemic stroke, patients during the acute stage of ischemic stroke, also received other treatments like antiplatelet therapy and/or cerebral circulatory agents, which are the mainstay treatment for ischemic stroke. Therefore, HBOT may not show its adjunctive effect significantly in mild stroke. Four, the patients included in this study are of mild severity. The degree of improvement for moderate-to-severe stroke may be more obvious than that of mild disease. 

This study has several limitations. First, although the case number is small, increasing study number may show the more significant results. Second, outcome evaluation is merely with the NIHSS score; however, it may not completely represent functioning status [22]. Even though all the patients in the HBOT group could perform daily activities independently one month after treatment, more objective scales or examination techniques may be necessary for outcome evaluation. Third, patients are only followed-up until one month after HBOT. Therefore, long-term efficacy evaluation may be necessary.

## 5. Conclusions

The present study of HBOT with 10 repetitions at 2 ATA 100% oxygen for 60 minutes appears to be effective for patients of mild acute ischemic stroke. It also appears as a safe and harmless adjunctive treatment. However, because of the limitations described above, the effects of HBOT for acute ischemic stroke cannot be completely revealed and require more large-scale trials with carefully planned investigation. The protocol here set up a pilot study for future efforts about adjuvant stroke treatment by HBOT.

## Figures and Tables

**Table 1:** Baseline characteristics of the study participants.

	HBOT group (*n* = 16)	Controls (*n* = 30)	*P* value
Gender, male/female	13/3	15/15	0.550
Age (years)	68.375 (32–88)	68.867 (44–86)	0.896
Past history	1.75 (0–3)	1.7 (0–3)	0.930
Triglycerol (mg/dl)	137.375 (52–408)	165.138 (50–359)	0.602
T. cholesterol (mg/dl)	181.313 (116–290)	201.833 (122–282)	0.176
Homocysteine (*μ*mol/L)	11.54 (4.78–16.36)	11.27 (3.10–20.45)	0.122

Clinical characteristics			
Risk factor, yes/no	3/13	14/16	0.569
Aspirin, yes/no	12/4	25/5	0.698
Anticoagulant, yes/no	4/12	9/21	0.245
Piracetam, yes/no	11/5	13/17	0.100
Antilipid agents, yes/no	4/12	10/20	0.063
Antihypertensives, yes/no	13/3	25/5	0.100
Oral antidiabetics, yes/no	7/9	11/19	0.100
Rehabilitation, yes/no	12/4	21/7	0.516
